# Terahertz Imaging for Breast Cancer Detection in Animal Models: A Literature Review with Narrative Synthesis

**DOI:** 10.3390/medsci14020323

**Published:** 2026-06-15

**Authors:** Maria Elena Niţă, Daniela Roxana Matasariu, Mioara Calipsoana Matei, Ana Cazacu, Bogdan Ionel Tamba, Delia Ciobanu Apostol, Cătălin Borcia, Cristina Mariana Uritu, Mitica Ciorpac, Alexandra Ursache, Cristina Elena Mandici, Cristina David, Radu Dănilă, Mihaela Baican, Vlad Ghizdovăț, Irena Cristina Grierosu, Cipriana Ștefănescu

**Affiliations:** 1 Grigore T. Popa University of Medicine and Pharmacy Iasi, 700115 Iasi, Romania; nita.maria-elena@d.umfiasi.ro (M.E.N.); mioara.matei@umfiasi.ro (M.C.M.); bogdan.tamba@umfiasi.ro (B.I.T.); delia.ciobanu@umfiasi.ro (D.C.A.); alexandra.ursache@umfiasi.ro (A.U.); cristina-elena.mandici@umfiasi.ro (C.E.M.); cristina.david@umfiasi.ro (C.D.); radu.danila@umfiasi.ro (R.D.); mihaela.baican@umfiasi.ro (M.B.); vlad.ghizdovat@umfiasi.ro (V.G.); irena.raileanu@umfiasi.ro (I.C.G.); cipriana.stefanescu@umfiasi.ro (C.Ș.); 2 Cuza Voda Clinical Hospital Obstetrics and Gynecology Iasi, 700038 Iasi, Romania; 3 Dr. Iacob Czihac Military Emergency Clinical Hospital Iasi, 700483 Iasi, Romania; 4 Department of Exact Sciences, Ion Ionescu de la Brad Iasi University of Life Sciences, 700490 Iasi, Romania; ana.cazacu@iuls.ro; 5 Advanced Research and Development Centre for Experimental Medicine “Prof. Ostin C. Mungiu”—CEMEX, Grigore T. Popa University of Medicine and Pharmacy Iasi, 700490 Iasi, Romania; cristina-mariana.uritu@umfiasi.ro (C.M.U.); mitica.ciorpac@umfiasi.ro (M.C.); 6 St. Spiridon University Hospital, 700111 Iasi, Romania; 7 Faculty of Physics, Alexandru Ioan Cuza University, 700506 Iasi, Romania; cborcia@uaic.ro; 8 IMAGO-MOL Cluster Iasi, 700115 Iasi, Romania

**Keywords:** terahertz imaging, breast cancer, diagnosis, detection features, animal model

## Abstract

**Background and Objectives:** Breast cancer remains one of the most common malignancies worldwide, and early detection plays a crucial role in improving treatment outcomes and reducing mortality. Several experimental studies using animal models of breast cancer have explored the potential of terahertz-based technologies in this field. However, their preclinical evidence base in breast cancer remains heterogeneous and has not been systematically synthesized with a focus on experimental models, imaging protocols, and barriers to translation. **Methods:** We conducted a descriptive systematic review, according to PRISMA guidelines, of 10 articles selected from a total of 372 identified across four databases—PubMed, Embase, Web of Science, and Cochrane—regarding the diagnostic performance of terahertz (THz) imaging in breast cancer animal models. We included studies that used rodent models diagnosed with breast cancer, subsequently confirmed through histological examination, and extracted relevant data. **Results:** The results were synthesized using a narrative approach. Most studies used C57BL/6J mice with E0771 cell line-induced breast tumors, with histopathology as the reference standard. In the reflection mode, at frequencies between 0.1 and 4 THz, the identification of tumoral, fibrous, fat, and muscle tissues was possible. **Conclusions:** Overall, the available preclinical evidence supports THz imaging as a promising proof-of-concept approach for breast tissue characterization, but not yet as a standardized or clinically translatable diagnostic platform. Future studies should use harmonized animal models, standardized acquisition and specimen-handling protocols, transparent reporting of classification workflows, and consistent outcome metrics to enable comparison across studies and to clarify the biological and biophysical determinants of THz contrast in breast cancer.

## 1. Introduction

Cancer represents a complex genetic disorder characterized by the disruption of DNA repair processes and remains one of the leading causes of death worldwide, according to the World Health Organization [1]. Gaps in women’s health research have often led to a limited understanding of female biology. Personalized, cost-effective, and reliable emerging technologies have the potential to address these concerns [2]. In this context, breast cancer—described as one of the “invisible threads” affecting women [3]—is the leading cause of cancer-related death among women [4,5].

Despite advances in current diagnostic methods, the early-stage detection of this disease remains challenging due to its heterogeneity and the lack of specific genomic biomarkers [6,7]. Currently available diagnostic approaches include clinical examinations and various imaging techniques, which may be structural (mammography, CT, MRI), functional, or hybrid (PET-CT, SPECT-CT), each with its specific limitations and contraindications [8,9,10]. However, most of these methods detect tumors only once clinical manifestations are present [11,12,13], despite significant improvements in spatial resolution, sensitivity, examination time, and radiation exposure [14].

Most women diagnosed with breast cancer undergo breast-conserving surgery, which involves the excision of tumor tissue with negative resection margins. In approximately 15–20% of cases, complete tumor removal with adequate margins is not achieved, increasing the risk of recurrence and consequently reducing survival rates [9,15,16]. Furthermore, although tissue biopsy remains the gold standard for diagnosis, it is important to note that it is still a time-consuming method (Table 1).

**Figure 1:**
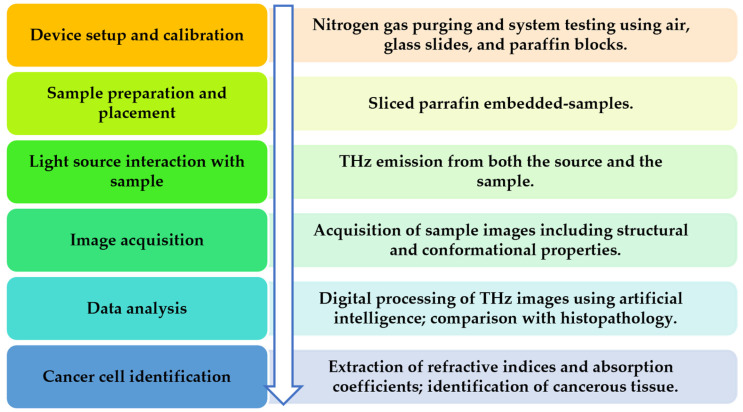
Terahertz working protocol (adapted from Vohra, 2020 [9] and Ferdous, 2024 [23]).

Terahertz (THz) radiation is part of the electromagnetic spectrum, located between the microwave and infrared regions, thus bridging the gap between electronics and photonics and exhibiting properties of both. Microwaves possess good penetration capabilities in many non-metallic materials but lack the ability to accurately distinguish between different tissue types. In contrast, light waves interacting with a material exhibit specific reflection, absorption, and penetration properties, contributing to their characteristic signature [18]. In this context, THz imaging has been proposed as a potential technique for tumor identification [19], based on variations in tissue water content during disease progression, resulting from increased vascularization or the presence of edema [6], as well as increased cancer cell metabolism. This enables the accurate identification of lesion-specific THz absorption peaks [20], with a reported sensitivity (80%) and specificity (82%) [21]. Due to its non-ionizing and non-invasive nature [22], THz technology represents an attractive and innovative, yet still incompletely studied, imaging modality for disease detection and evaluation, as well as for patient monitoring and follow-up in the context of personalized treatment [20]. Despite the growing number of publications in recent decades, primarily focused on technological advancements toward cost-effective devices, there remains a critical need for standardized operating protocols (Figure 1) and improved processing of digitally acquired images.

Preclinical models of carcinogen-induced breast tumors in rodents have been widely used by various research groups to mimic and better understand the relationship between cancerous cells and surrounding tissue types, thereby improving tissue differentiation [24]. As most studies employed mice—either C57BL/6 black laboratory mice [25,26,27,28,29] or BALB/cAnN.Cg-Foxn1 nu/CrlNarl mice [30,31]-the most commonly used cell line for breast cancer induction was E0771 [25,26,27,28,29], followed by MDA-MB-231 [30,31]. Additionally, a transgenic mouse model of breast cancer—mouse mammary tumor virus–polyoma middle T antigen (MMTV-PyMT)—has been used due to its similarity to the complexity and progression of human breast cancer. Sprague–Dawley rats have also been shown to develop mammary tumors between 50 and 155 days following injection with N-ethyl-N-nitrosourea (ENU), representing another experimental model [24].

To our knowledge, no descriptive systematic review has been published to date regarding the diagnostic performance and technical aspects of THz imaging in breast cancer animal models. The main objectives of this study were to evaluate the ability of THz imaging to identify and differentiate cancerous tissues in animal models of breast cancer, using histopathological examination as the reference standard, and to analyze the experimental conditions and technical parameters reported in the included studies (e.g., imaging modality, frequency range, experimental setup, and sample preparation) in order to identify patterns. The ultimate aim of this review is to address existing gaps in the field and to support the development of a standardized protocol suitable for future multicentre studies.

## 2. Results

After applying the predefined inclusion and exclusion criteria, the study selection consisted of a total of 10 articles.

Studies were grouped into four main categories according to the type of THz imaging technology used, animal model characteristics (in accordance with the ARRIVE guidelines), experimental setting (in vivo or ex vivo), and reported outcome measures. A tabulation and thematic comparison were applied to identify methodological trends and diagnostic performance patterns.

### 2.1. Animal Model Characteristics

Regarding the number of animal groups used in the included studies, this ranged from 2 to 20, also depending on the number of samples obtained from each rodent, with most studies utilizing two halves of the same sample (Table 2).

As for the comparison methods used to standardize the THz method, nine out of ten studies used histopathology as the gold standard reference method, whereas one study used both histopathology and MRI. Chen et al. (2021) estimated the detection limit to be less than 1 mm^3^ and demonstrated improved tissue penetration of the system at thicknesses of up to 8 cm, using a highly sensitive Schottky diode operated at cryogenic temperatures [31]. In that study, no external validation was required for the THz method [31]. It is important to note that the current detection limit of X-ray mammography is approximately 2 mm in diameter (Table 2).

Regarding the tumor induction models used, six out of ten studies used E0771 adenocarcinoma cell lines (with tumor dimensions reaching up to 1 cm for testing purposes), compared with two studies that used MDA-MB-231 cells (with tumor diameters of approximately 4 mm). In contrast, one study used N-ethyl-N-nitrosourea for tumor induction (tumor diameters ranging from 8 to 18 mm). Another study used the MMTV-PyMT model (mouse mammary tumor virus long terminal repeat–driven polyoma middle T antigen), resulting in the development of multifocal adenocarcinomas within the mammary epithelium and metastatic lesions; the tumor size did not exceed 2.5 cm (Table 2) [32].

The most frequently used species in the analyzed studies was the C57BL/6J mouse fed a high-fat diet, reaching a body weight of approximately 35 g. Two studies performed experiments on 4- to 6-month-old female BALB/cAnN.Cg-Foxn1 nu/CrlNarl mice, which carry a genetic mutation resulting in a reduced or absent thymus. Another study used 30-day-old female Sprague Dawley rats with a target weight of 150–186 g. It should also be noted that El-Shenawee et al. (2019) and Vohra et al. (2018) conducted experiments on transgenic mouse models (Table 2) [28,32].

### 2.2. Technical Aspects in the Included Studies

The most commonly used THz frequency range was 0.1–4 THz for sample scanning, while some studies employed lower frequencies of 108–143 GHz. It is also important to highlight that Liu et al. (2022) [29] used a more limited frequency range between 0.5 and 1 THz for tissue recognition (Table 3).

Regarding the devices used, eight studies performed experiments using a TPS Spectra 3000 (Manufacturer: TeraView Ltd., Cambridge, UK) THz spectrometer, with the following specifications: a 780 or 800 nm Ti:sapphire laser, a GaAs photoconductive antenna, and a 200 μm step-size motor. Two studies used a THz source based on a YIG oscillator module and a cryogenically cooled Schottky diode detector. In addition, most studies used the reflection mode, while two used the transmission mode (Table 3). The reflection mode, utilizing pulsed THz, provided richer information due to the possibility of acquiring 2D/planar images that could be compared to gold-standard existing methods, in contrast with the transmission mode that could obtain only a single spectrum for each point of the scanned tissue.

As for the experimental protocol, all studies initially analyzed fresh samples using THz imaging, followed by analysis of paraffin-embedded samples, and subsequently by histopathological examination (Table 3). An important aspect to mention is that due to shape and size alterations in the paraffin-embedded samples, a 50% reasonable correlation between THz and pathology was observed.

### 2.3. Experimental Setting

Regarding tumor cell line inoculation, several methods were used, including the direct injection of cells into the mammary fat pad or into fat deposits, intraperitoneal injection (lower right abdominal quadrant), and implantation into the dermal layer of mouse skin. All procedures were performed under anesthesia to minimize animal discomfort and to comply with ethical regulations (the ARRIVE guidelines), resulting also in multiple scans of the same specimen to reduce the number of animals used during these procedures and to help with the protocol standardization process.

For sample preparation, most studies used thin, fresh tissue sections placed between two polystyrene plates. Samples were subsequently embedded in paraffin and further analyzed using THz imaging and histopathological techniques to confirm tumor location, diagnosis, and extent. In one study, the embedded dorsal tissue was placed between two glass coverslips for THz analysis (Table 4).

Regarding potential sources of error, several limitations were identified, including the inability to replicate connective tissues other than fat, difficulties in obtaining realistic models for accurate structural identification, and reduced detection sensitivity in fresh tissue due to differences in imaging surfaces. Additional limitations include artifacts caused by fluid-filled necrotic regions (internal mismatch after mesh morphing), difficulties in differentiating muscle, skin, and salivary gland tissues, the loss of tissue heterogeneity, and challenges in shape comparison due to dehydration-induced deformation between fresh tissue and histopathological samples. Further issues include edge effects in pixel classification near boundaries, difficulties in preserving outer contour integrity, and the misclassification of non-tissue-related artifacts (Table 4).

### 2.4. Classification Methods

The image classification process represents a distinct research area. Advances in computer science over the past decade have also been extended to THz imaging, where artificial intelligence and its subfields—including machine learning and natural language processing—have been applied to support clinical diagnosis and outcome prediction, such as tumor detection and characterization [34].

The machine learning algorithms used in the selected studies include both supervised and unsupervised approaches, as listed below: Bayesian mixture models (unsupervised Gaussian and t-distribution components, and supervised regression-based approaches), Markov chain Monte Carlo (MCMC) algorithms for pathology-based morphing; supervised multinomial Bayesian learning, polynomial regression, and kernel regression models; expectation-maximization (EM)-based unsupervised classification for mesh morphing; wavelet synchro-squeezed transform methods (WSST); convolutional neural networks (CNNs) applied in the 0.1–3 THz range; additional expectation-maximization techniques; and Delaunay triangulation (Table 5).

Due to the heterogeneity of the included studies and missing data, a quantitative performance comparison of automated classification algorithms was not possible. Nevertheless, the Markov chain Monte Carlo (MCMC) algorithm appears to be the most frequently used, as it was reported in four of the included studies, succeeding in identifying and differentiating cancerous, fat and muscular tissues.

**Figure 2:**
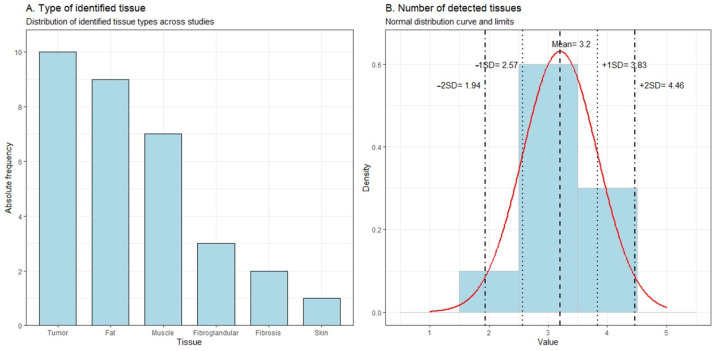
Distribution of identified tissue types across studies (the red line represents the theoretical normal (Gaussian) distribution, while the bars represent the observed distribution; The method was able to discriminate between 2 and 5 tissue types, with a mean of 3 tissue types).

There are some discrepancies in shape deformation, misalignment, and edge recognition [33], as well as in the classification of non-tissue-related artifacts or even in tissue differentiation, depending on the algorithms used [29], as shown in Table 6 and Figure 2. We can observe that from Figure 2 that most studies analyzed and better identified the following tissues: fat, muscle, and tumor.

### 2.5. Quality Assessment of Included Studies

The *SYRCLE* risk-of-bias assessment (Systematic Review Centre for Laboratory Animal Experimentation) indicated that most included studies had an unclear risk of bias across several domains, particularly with respect to randomization, allocation concealment, and blinding procedures.

According to the *Kmet* checklist, scores were interpreted as follows: >0.80–strong quality, 0.70–0.79–good quality, 0.50–0.69–fair quality, and <0.50–poor quality. The included studies demonstrated several strengths, including clearly defined objectives, appropriate experimental designs, and well-described THz imaging protocols. Common limitations included the lack of formal sample size justification and the limited reporting on the control of environmental confounders [35].

Overall, most studies scored within the moderate range (0.73–0.77), while three out of ten studies achieved high scores (0.81), as shown in Table 7. These findings indicate the overall moderate methodological quality of the included studies.

## 3. Discussion

**Figure 3:**
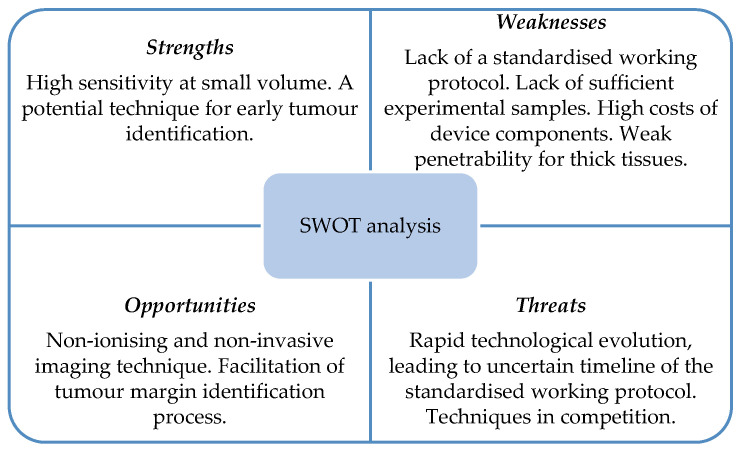
SWOT analysis of terahertz imaging.

Our review suggests that, under controlled conditions, THz imaging can indeed highlight cancerous regions and their margins in breast tissue, consistent with the authors’ goals, enabling differentiation and facilitating accurate diagnosis to improve breast cancer treatment outcomes. In recent decades, THz imaging has emerged as a diagnostic method in clinical practice, through preclinical studies, due to its non-invasive and non-contact properties, showing potential to simplify the process of establishing a personalized diagnosis. This technique has been reported as safer and more accurate than X-rays or MRI (Figure 3) [18].

### 3.1. Factors Influencing THz Diagnostic Performance

**Figure 4:**
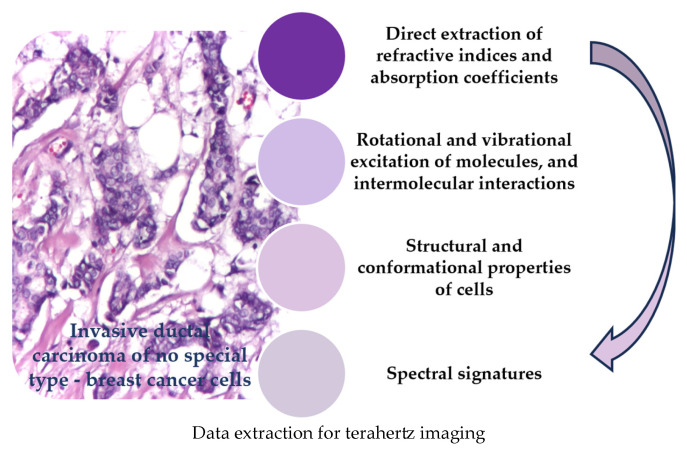
THz identification characteristics of breast cancer cells-human histopathological sample of invasive ductal carcinoma of no special type, haematoxylin and eosin (H&E) staining, 20× magnification, and data type extraction for terahertz imaging.

The main descriptive parameters of THz analysis include transmission, reflection, and absorption. Their photon energy lies within the range of hydrogen bonding, charge-transfer interactions, and van der Waals forces, which makes THz radiation particularly sensitive to molecular and intermolecular dynamics. As for the interaction with biological tissues, small and simple molecules may exhibit characteristic absorption in this range. In contrast, larger molecules present multiple and unique vibrational modes due to complex intramolecular interactions, enhancing molecular-level recognition and defining their spectral signatures (Figure 4). The primary contrast agent in THz imaging is water. On the one hand, this provides a practical advantage in cancer tissue imaging, due to tissue heterogeneity (e.g., swelling and scarring) and variations in hydration. On the other hand, water acts as a masking medium in the spectroscopy of other biomolecules of interest [36]. Consequently, fresh tissue is better analyzed using reflection imaging, whereas dehydrated (fixed) cancer tissue can be imaged in both the reflection and transmission modes [37].

The purpose of THz imaging was to achieve an accurate description and reconstruction of the object under study. Each wave peak corresponds to a different point on the object surface; thus, layer thickness can be estimated from the time delay of THz pulses, while pulse amplitude, shape, and temporal delays provide contrast information [18]. Consequently, Okada et al. (2022) successfully visualized an unstained breast cancer lesion of 1 mm using both the transmission and reflection modes, and were even able to identify a necrotic region of approximately 500 μm within the lesion [38]. Image detection represents only the first step in image acquisition, whereas image reconstruction remains more challenging, requiring various algorithms and digital processing techniques [18].

In the reviewed studies, we extracted pathognomonic characteristics of breast cancer from animal models to facilitate tumor differentiation and identification using THz imaging, using the gold-standard diagnostic method-histopathology, as a comparator. As such, there is an ongoing need for systematic reviews to identify research gaps in order to find patterns that can lead to quicker identification of the tumor tissue [39].

Our analysis highlighted that in Chen et al. (2011), the detection limit was 0.05 mm^3^ in tissues thinner than 5 mm, whereas in Chen et al. (2021), it improved to approximately 1 mm^3^ for tissues up to 8 cm in depth [30,31]. It is important to note that in THz imaging, increased tissue thickness is associated with decreased sensitivity [33].

The differences between continuous-wave and pulsed-wave THz sources are primarily related to hardware configuration. In addition, pulsed THz imaging provides richer information due to time-domain acquisition and processing. Depth information can be retrieved by synchronizing the pulse, where the temporal position of the reflected peak—i.e., the time delay relative to the main pulse—can be converted into depth [18].

### 3.2. Challenges in Tissue Differentiation

Overall, several aspects of experimental design and image classification methods still require improvement to maximize information extraction and reduce potential errors.

Mesh morphing algorithms can correct complex deformations and reduce human bias, enabling improved classification of FFPE (formalin-fixed paraffin-embedded) tissue images in agreement with histopathological masks. A correlation of approximately 50% was reported between THz imaging and histopathology, partly due to fluid removal, which minimizes morphological distortions in sample shape and size [25,27,32]. Difficulties were encountered in differentiating between muscle and tumor, fat and tumor, skin and tumor, fibro-glandular tissue and tumor, as well as in identifying salivary glands, often at the expense of accurate tumor identification [24,28,31,32]. Frequencies between 0.1 and 4 THz were used for sample scanning, while tissue recognition was typically performed in the range of 0.5 to 1 THz. In addition, selection bias was reduced by standardizing pixel sampling per region, allowing training on certain samples and testing on others to improve classification performance [26].

The inability to incorporate connective tissues other than fat may limit tumor identification in complex preclinical models and hinder the replication of human breast cancer developmental conditions for an accurate THz imaging assessment [27,30]. Another important limitation is the reduced detection accuracy in fresh tissue due to differences in imaging surfaces, as well as artifacts such as fluid-filled necrotic gaps (internal mismatch), all of which require further improvement for accurate cancer identification [26,27].

It is important to note that these preclinical models showed multiple similarities with human samples [24]; however, further validation on human tissue is still required before this method can be used for malignancy diagnosis. Multicenter studies on human pathology samples together with a standardized working protocol and an efficient infrastructure are still needed to validate this novel imaging technique, to improve the understanding of the biophysical mechanisms of the disease at the molecular level, and to provide a quantitative assessment of accuracy for subsequent image interpretation and classification.

### 3.3. Limitations of the Study

There are several limitations to be acknowledged in this systematic review.

First, the number of eligible studies was limited, reflecting the emerging nature of THz imaging in preclinical breast cancer research. In addition, many studies included small sample sizes, which may affect the robustness of the reported findings.

Second, a high degree of heterogeneity was observed across studies in terms of animal models, experimental design, THz system parameters, and outcome reporting. This variability limited comparability and precluded a meta-analysis.

Third, differences in tumor models, tissue preparation, and imaging protocols may have introduced variability in diagnostic performance, affecting the generalizability of the results.

Finally, despite a comprehensive search strategy, restricting inclusion to English-language publications may have introduced language bias.

Therefore, the findings should be interpreted with caution, and further standardized, large-scale preclinical studies are needed to better define the diagnostic potential of THz imaging and to establish a unified working protocol.

## 4. Materials and Methods

This systematic review with a narrative synthesis aimed to analyze published data on the diagnostic performance and experimental characteristics of THz imaging in animal models of breast cancer and was conducted between 2005 and 2026.

The review was conducted and reported in accordance with the PRISMA-DTA (Preferred Reporting Items for Systematic Reviews and Meta-Analyses of Diagnostic Test Accuracy Studies) guidelines. The study protocol was registered in PROSPERO, the international prospective register of systematic reviews (registration number CRD420261323794, March 2026).

### 4.1. Study Design

The review methodology was based on the PICO framework, defined as follows: population—rodent models of breast cancer, including chemically induced, cell line-induced, or genetically engineered models; intervention—the application of THz imaging or spectroscopy for tissue characterization; comparator—histopathological examination as the reference standard; and outcome—diagnostic performance indicators, including sensitivity, specificity, and tissue differentiation capability.

Due to the anticipated heterogeneity in study design, animal models, and reported outcomes, a quantitative meta-analysis was not considered appropriate. Therefore, a structured narrative synthesis approach was adopted to summarize the findings.

In addition to diagnostic performance, the review aimed to identify commonly used experimental models and technical parameters of THz systems, to highlight methodological trends and factors influencing the performance of this imaging modality in preclinical breast cancer research.

### 4.2. Screening Strategy

A systematic search was conducted in four electronic databases: PubMed, Embase, Web of Science, and the Cochrane Library, yielding a total of 372 records.

The search strategy combined controlled vocabulary terms (e.g., MeSH) and free-text keywords related to breast cancer and THz imaging. The main search terms included “breast cancer,” “breast neoplasms,” “terahertz imaging,” “Terahertz imaging,” “diagnosis,” “detection,” and “animal models.” Boolean operators (AND/OR) were used to combine search terms, and the strategy was adapted for each database.

Only studies published in English were included. Grey literature was searched using Google Scholar. Additionally, the reference lists of all included studies and relevant review articles were manually screened to identify further eligible studies.

All records were imported into Zotero, version 7.0.32 (64-bit), where duplicates and ineligible publication types (conference papers, book chapters, and retracted articles) were removed.

Study selection was performed independently by two reviewers (MEN and DRM). Titles and abstracts were screened to identify potentially relevant studies, followed by a full-text assessment based on predefined inclusion and exclusion criteria and subsequent data extraction. Any discrepancies were resolved through discussion or, when necessary, by consultation with a third reviewer (CS).

**Figure 5:**
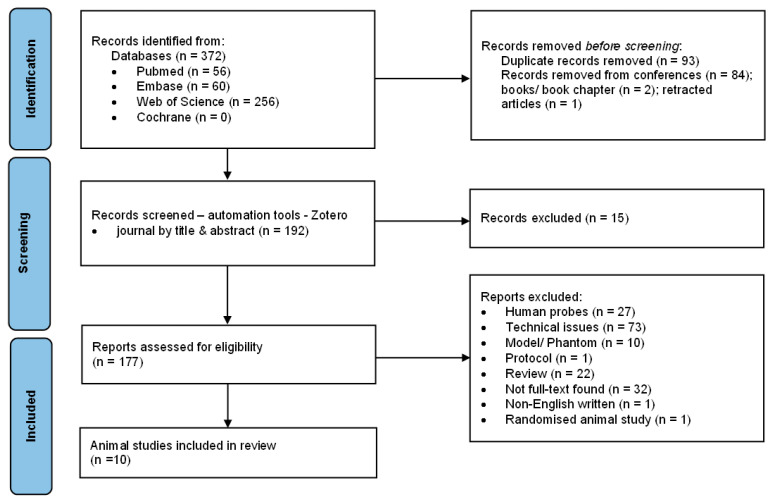
PRISMA 2020 flow diagram of study selection (preferred reporting items for systematic reviews and meta-analyses).

The study selection process and the search queries used for carrying out this review are presented in a PRISMA flow diagram and in the table below (Figure 5, Table 8).

### 4.3. Inclusion Criteria

Eligible studies met the following criteria: studies involving rodent models of breast cancer with histopathological confirmation. Only peer-reviewed original research articles were included, in vivo or ex vivo laboratory studies, diagnostic accuracy studies, and controlled experimental designs evaluating the application of THz imaging for breast cancer detection or tissue differentiation.

### 4.4. Exclusion Criteria

The following types of studies were excluded:

- Human studies on breast cancer;
- Studies based on phantom or synthetic models;
- Studies available only as abstracts without full-text access;
- Conference papers due to insufficient methodological detail;
- Editorials, commentaries, and review articles;
- Simulation-only studies without biological or experimental validation;
- Study protocols without experimental results;
- Studies not related to breast cancer applications;
- Purely technical studies without biological validation;
- Duplicate publications;
- Articles with no full-text available;
- Studies published in languages other than English.

The following information was extracted from each study:

(a) Study characteristics:
  - Author(s);
  - Year of publication;
  - Study design.
(b) Animal model characteristics:
  - Species;
  - Number of animals;
  - Tumor induction method (e.g., cell-line inoculation or genetically engineered models);
  - Cell lines used.
(c) Intervention characteristics:
  - Type of THz imaging system used;
    ○ Frequency range or spectral characteristics;
  - Experimental or measurement conditions;
  - Type of tissue analyzed.
(d) Outcome measures:
  - Tissue differentiation capability;
  - Classification performance;
  - Diagnostic performance indicators (e.g., sensitivity, specificity, accuracy, when reported);
  - Detection limits.

### 4.5. Quality Assessment

Assessment of publication bias was not performed due to the expected limited number and heterogeneity of the included preclinical studies.

Risk of bias was assessed using the *SYRCLE* Risk of Bias Tool (Systematic Review Centre for Laboratory Animal Experimentation, Utrecht, The Netherlands), which was specifically developed for animal intervention studies. Two reviewers (MEN and DRM) independently evaluated each study across the following domains: sequence generation, baseline characteristics, allocation concealment, random housing, blinding of caregivers, random outcome assessment, blinding of outcome assessors, incomplete outcome data, selective reporting, and other sources of bias. Each domain was rated as low, high, or unclear risk of bias. Any disagreements were resolved through discussion or consultation with a third reviewer (MCM).

In addition, the reporting quality was assessed using the *Kmet* Standard Quality Assessment Criteria to provide a complementary evaluation. Each item was scored as no = 0, partial = 1, and yes = 2, and a summary score was calculated for each study.

## 5. Conclusions and Future Directions

This descriptive review indicates that THz imaging has promising preclinical utility for breast cancer tissue characterization in rodent models, particularly for differentiating tumor-containing regions from surrounding non-tumor tissue under controlled experimental conditions. Across the included studies, however, the evidence base was limited by substantial heterogeneity in animal models, THz platforms, acquisition parameters, tissue preparation methods, analytical workflows, and outcome reporting.

Reported performance appears to depend not only on tumor presence, but also on specimen hydration, tissue thickness, fixation state, deformation during histopathological processing, and the choice of computational classification method. Recurrent challenges included limited penetration depth, mixed-tissue interfaces, edge artifacts, and inconsistent differentiation between the tumor and adjacent muscle, fat, skin, or fibroglandular tissue.

Future studies should prioritize standardized experimental protocols, transparent reporting of acquisition and preprocessing steps, the external validation of classification pipelines, and harmonized outcome measures to improve the obtained results. Comparative work across xenograft, chemically induced, and genetically engineered models will also be important for clarifying which biological and microenvironmental features most strongly influence THz contrast. Taken together, the current literature supports the continued preclinical development of THz-based breast tissue imaging, but stronger and more standardized evidence is required before its translational role can be defined with confidence.

## Figures and Tables

**Table 1:** Comparison of diagnostic imaging techniques (adapted from Chen, 2026 [17]).

Index	Terahertz Imaging	Ultrasound Imaging	CT	Mammography	PET-CT	MRI
Penetration depth	<1 mm (in vivo, aqueous tissue); up to several cm (ex vivo, dehydrated samples)	Several cm	Whole body	Whole body	Whole body	Whole body
Detection time	Milliseconds	Minutes	Minutes	Seconds	Hours	Minutes
Frequency/wavelength	0.1–10 THz/30 μm–3 mm	1–20 MHz	100 kHz–100 MHz	10^16^–10^19^ Hz/0.01–10 nm	511 keV γ-rays	~10–300 MHz/~1 m–1 mm
Specificity/accuracy	Medium (molecular signatures)	Low (structural)	Medium (density-based)	High (dense tissue contrast)	High (metabolic activity)	High (for soft tissue)
Imaging process	Identification of intracellular water content and metabolic processes	Image derived from echo return time and intensity	X-ray attenuation–3D imaging	X-ray attenuation–2D imaging	Measurement of radionuclide uptake	Proton detection; depending on relaxation times (T1, T2)
Key findings	Non-ionizing; Water-sensitive; Fingerprint spectra	Real-time; Requires coupling agent	High anatomical resolution; Ionizing radiation	Ionizing radiation	Functional imaging; Requires radiotracer	Non-ionizing; Water and microenvironment sensitive; Multiparametric capability

THz–terahertz; MHz–Megahertz; CT–Computed tomography; PET-CT-Positron emission tomography–computed tomography; MRI–Magnetic resonance imaging.

**Table 2:** Characteristics of the included rodent species.

Year of Publication	Authors	Sample Size (Number of Animals/Samples)	Species	Tumor Cell Type Used	Species Characteristics
2011	Chen et al. [30]	10	Mouse	MDA-MB-231	BALB/cAnN.Cg-Foxn1 nu/CrlNarl
2018	Bowman et al. [27]	10	Mouse	E0771	C57BL/6
2018	Chavez et al. [25]	13	Mouse	E0771	C57BL/6
2018	Vohra et al. [32]	4	Transgenic mouse (MMTV-PyMT)	No tumor cell induction needed	Spontaneous breast tumors
2019	El-Shenawee et al. [28]	N/A	Human/mouse	E0771	MMTV-PyMT, C57BL/6, human samples
2021	Chavez et al. [26]	6	Mouse	E0771	C57BL/6
2021	Chen H. et al. [31]	20	Mouse	MDA-MB-231	BALB/cAnN.Cg-Foxn1 nu/CrlNarl
2021	Vohra et al. [24]	20	Rat	N-ethyl-N-nitrosourea	Sprague Dawley
2022	Liu et al. [29]	11	Mouse	E0771	C57BL/6
2025	Cong et al. [33]	N/A	Mouse/Cell cultures	E0771	C57BL/6

N/A–not available; MMTV-PyMT-mouse mammary tumor virus-polyoma middle T antigen; MDA MB 231 and E0771–breast cancer cell-line cultures; BALB/cAnN.Cg-Foxnlnu/CrlNarl and C57BL/6–mouse species used in experiments.

**Table 3:** Technical characteristics of THz devices.

Year of Publication	Authors	Terahertz System Type	THz Device	Step Size (μm)	Frequency Range (THz)
2011	Chen et al. [30]	Transmission imaging system	YIG oscillator module (THz Source)	N/A	108 × 10^−3^– 143 × 10^−3^
2018	Bowman et al. [27]	Reflection mode, pulsed THz	TPS Spectra 3000 (TeraView); Ti:sapphire laser 800 nm	200	0.1–4
2018	Chavez et al. [25]	Reflection mode, pulsed THz	TPS Spectra 3000 (TeraView); Ti:sapphire laser 800 nm	200	0.1–4
2018	Vohra et al. [32]	Reflection mode, pulsed THz	800 nm wavelength laser beam, GaAs photoconductive antenna	200	0.1–4
2019	El-Shenawee et al. [28]	Reflection mode, pulsed THz	TPS Spectra 3000 (TeraView); Ti:sapphire laser 800 nm	200	0.1–4
2021	Chavez et al. [26]	Reflection mode, pulsed THz	TPS Spectra 3000 (TeraView); Ti:sapphire laser 780 nm	200	0.1–4
2021	Chen H. et al. [31]	Transmission imaging system	YIG oscillator module (THz source)	N/A	108 × 10^−3^– 143 × 10^−3^
2021	Vohra et al. [24]	Reflection mode, pulsed THz	780 nm wavelength laser beam, GaAs photoconductive antenna	200	0.1–4
2022	Liu et al. [29]	Reflection mode, pulsed THz	TPS Spectra 3000 (TeraView); Ti:sapphire laser 800 nm	200	0.5–1
2025	Cong et al. [33]	Reflection mode, pulsed THz	780 ±10 nm wavelength laser beam, GaAs photoconductive antenna	N/A	0.1–4

THz–terahertz; TPS–TeraView spectrometer system (TeraPulse system); GaAs–Gallium Arsenide.

**Table 4:** Experimental setting of the included studies.

Year of Publication	Authors	Diameter of Extracted Probe (mm)	Inoculation Method	Tissue Type	Working Conditions	Duration of Experiment (min)
2011	Chen et al. [30]	4	Subcutaneous xenograft; fatty tissue implantation on day 6 with tumor cells	Tumor, fat, skin	Room temperature (23 °C), 50% humidity	5
2018	Bowman et al. [27]	10	Injected into flank as subcutaneous bolus	Tumor, fat, muscle	N/A	N/A
2018	Chavez et al. [25]	10	Injected into fat pads	Tumor, fat, muscle (paraffin-embedded)	N/A	N/A
2018	Vohra et al. [32]	25	No inoculation required	Tumor, fibrous tissue, fat, muscle	Imaging performed 1 h after excision	N/A
2019	El-Shenawee et al. [28]	10	N/A	Tumor, fat, fibroglandular tissue	Room temperature	30–40
2021	Chavez et al. [26]	10	N/A	Tumor, fibrous tissue, muscle, fat	System purged with dry nitrogen gas for 30 min prior to imaging	N/A
2021	Chen H. et al. [31]	N/A	Subcutaneous xenograft; fatty tissue implantation on day 7 with tumor cells	Tumor, fat, skin	Each mouse scanned three times	1
2021	Vohra et al. [24]	8–18	Intraperitoneal injection (lower right abdominal quadrant)	Tumor, fat, fibroglandular tissue, muscle	Animals aged 9–21 weeks at extraction	35
2022	Liu et al. [29]	10	Injection into subcutaneous breast fat pad	Tumor, fat, muscle	N/A	N/A
2025	Cong et al. [33]	10	Injection into subcutaneous breast fat pad	Tumor, fibroglandular tissue	N/A	N/A

N/A–not available.

**Table 5:** Machine learning methods for image classification.

Year of Publication	Authors	Automated Learning Algorithms	Algorithm Performance
2011	Chen et al. [30]	N/A	N/A
2018	Bowman et al. [27]	Bayesian unsupervised algorithm for tissue classification (cancer, fat, muscle)–Markov chain Monte Carlo (MCMC)	N/A
2018	Chavez et al. [25]	Bayesian unsupervised algorithm for tissue classification (cancer, fat, muscle)–Markov chain Monte Carlo (MCMC)	N/A
2018	Vohra et al. [32]	N/A	N/A
2019	El-Shenawee et al. [28]	Image- and spectrum-based classification algorithms; automated learning approaches for classification	Good performance in tissue differentiation; strong discrimination between cancerous and healthy tissue
2021	Chavez et al. [26]	Polynomial and kernel regression models; Markov chain Monte Carlo (MCMC)	Improved performance compared to other methods
2021	Chen H. et al. [31]	N/A	N/A
2021	Vohra et al. [24]	Expectation-Maximization (EM) algorithm and Markov chain Monte Carlo (MCMC)	N/A
2022	Liu et al. [29]	Preprocessing with WSST (wavelet synchro-squeezed transform) and classification using CNNs (convolutional neural networks) and Siamese networks	N/A
2025	Cong et al. [33]	N/A	High efficiency; strong differentiation between cancerous and normal cells

N/A–not available; WSST-wavelet synchro-squeezed transformation; CNN–convolutional neural networks.

**Table 6:** Summary of results from the included studies.

Year of Publication	Authors	Observations/Commentary	Technical Difficulties/Errors
2011	Chen et al. [30]	Early stage cancer detection with high sensitivity; demonstrated that THz imaging can differentiate tumors from adipose tissue and skin in animal models (not humans)	Limited penetration depth; high absorption due to water content; need for calibration
2018	Bowman et al. [27]	Improved classification of FFPE tissue images in agreement with pathology masks due to fluid removal; same laboratory and research group	Reduced detection in fresh tissue; appearance of fluid-filled necrotic gaps; difficulty differentiating muscle tissue; shape deformation
2018	Chavez et al. [25]	Mesh morphing algorithm used for evaluation and alignment	Resolution mismatch, shape deformation, misalignment
2018	Vohra et al. [32]	Histopathological processing altered tissue shape and size-~50% correlation between THz imaging and pathology; same laboratory and research group	Presence of fluid artefacts; degradation of fibrous and fatty tissue
2019	El-Shenawee et al. [28]	Tumors excised from human and mouse samples and phantoms; challenges due to overlapping properties of fibro-glandular and cancerous tissue; tissue changes during histopathology; same laboratory and research group	Limited penetration depth; variability between samples
2021	Chavez et al. [26]	Bias reduction achieved by equal pixel sampling per region, reducing training imbalance; training and testing performed on separate samples; same laboratory and research group	Shape deformation; internal mismatch after mesh morphing; difficulty differentiating muscle tissue; fluid-filled luminal gaps
2021	Chen H. et al. [31]	THz measurements performed ex vivo and in vivo for volume estimation	Difficulty differentiating skin and tumor tissue; variability in skin thickness assumptions
2021	Vohra et al. [24]	Preclinical models showed similarity to human tumors; minimal misclassification at tumor edges; comparison of transgenic and xenograft mouse models	Differences between model types; same laboratory and research group
2022	Liu et al. [29]	Variability observed among xenograft samples	Difficulty distinguishing muscular tissue; edge recognition issues; shape deformation; non-tissue artefact classification
2025	Cong et al. [33]	Increased tissue thickness reduces sensitivity; meta-surface improves contrast in distinguishing cancer from fibroblasts (up to ~50% improvement); parameter variation in cell culture and simulations	Increased thickness reduces sensitivity; edge contour integrity issues

FFPE–formalin-fixed paraffin-embedded.

**Table 7:** Quality assessment of the included studies (adapted from Kmet, 2004 [35]).

No	Criterion/Article	Chen, 2011 [30]	Bowman, 2018 [27]	Chavez, 2018 [25]	Vohra, 2018 [32]	El-Shenawee, 2019 [28]	Chavez, 2021 [26]	Chen, 2021 [31]	Vohra, 2021 [24]	Liu, 2022 [29]	Cong, 2025 [33]
1	Question/objective clearly described	2	2	2	2	2	2	2	2	2	2
2	Appropriate study design	2	2	2	2	2	2	2	2	2	2
3	Methods of sample/subject selection described	2	2	2	2	2	2	2	2	2	2
4	Sample size justified	1	1	1	1	1	1	1	1	1	1
5	Outcome measures clearly defined	2	2	2	2	2	2	2	2	2	2
6	Outcome measures valid and reliable	2	2	2	2	2	2	2	2	2	2
7	Exposure/intervention clearly described	2	2	2	2	2	2	2	2	2	2
8	Confounders described	1	1	1	1	1	1	1	1	1	1
9	Confounders controlled	1	1	1	1	1	1	1	1	1	1
10	Blinding of outcome assessment	N/A	N/A	N/A	N/A	N/A	N/A	N/A	N/A	N/A	N/A
11	Appropriate statistical/analytical methods	2	2	2	2	2	2	2	2	2	2
12	Variance/error estimates reported	1	1	1	1	1	2	2	1	2	1
13	Results reported in sufficient detail	2	2	2	2	2	2	2	2	2	2
14	Conclusions supported by results	2	2	2	2	2	2	2	2	2	2
	Raw score	20	20	20	20	20	21	21	20	21	19
	Denominator	26	26	26	26	26	26	26	26	26	26
	Normalized score	0.77	0.77	0.77	0.77	0.77	0.81	0.81	0.77	0.81	0.73
	Quality	Moderate	Moderate	Moderate	Moderate	Moderate	High	High	Moderate	High	Moderate

**Table 8:** Search queries.

Database	Search Terms	Filters Applied	Date Searched	Hits
PubMed	(breast OR mammary) AND (cancer OR neoplasm OR tumor OR ductal carcinoma) AND terahertz AND (imaging OR detection OR diagnosis OR diagnostic procedure)	2005–2026	22 March 2026	56
Embase	(‘breast’ OR ‘mammary’) AND (‘cancer’ OR ‘neoplasm’ OR ‘tumor’ OR ‘ductal carcinoma’) AND ‘terahertz’ AND (‘imaging’ OR ‘detection’ OR ‘diagnosis’ OR ‘diagnostic procedure’)	2005–2026	22 March 2026	60
Web of Science	(breast OR mammary) AND (cancer OR neoplasm OR tumor OR ductal carcinoma) AND terahertz AND (imaging OR detection OR diagnosis OR diagnostic procedure) (All Fields)	2005–2026	22 March 2026	256
Cochrane Library	(breast OR mammary) AND (cancer OR neoplasm OR tumor OR ductal carcinoma) AND terahertz AND (imaging OR detection OR diagnosis OR diagnostic procedure) in All Text—(Word variations have been searched)	2005–2026	22 March 2026	0

## Data Availability

The original contributions presented in this study are included in the article. Further inquiries can be directed to the corresponding author.

## Abbreviations

The following abbreviations are used in this manuscript:

DNA	Desoxyribonucleic acid
THz	Terahertz
MHz	Megahertz
CT	Computed Tomography
MRI	Magnetic Resonance Imaging
PET-CT	Positron emission tomography—computed tomography
MMTV-PyMT	Mouse mammary tumor virus-polyoma middle T antigen
ENU	N-ethyl-N-nitrosourea
N/A	Not available
GHz	Gigahertz
TPS	Terapulse
GaAs	Gallium arsenide
WSST	Wavelet synchro-squeezed transformation
CNN	Convolutional neural networks
SYRCLE	SYstematic Review Centre for Laboratory animal Experimentation
FFPE	Formalin-fixed paraffin-embedded
PRISMA-DTA	Guidelines for Diagnostic Test Accuracy described in the Preferred Reporting Items for Systematic Reviews and Meta-Analyses
PICO	Population, intervention, comparison, outcome
